# Clinically Deployed Computational Assessment of Multiple Sclerosis Lesions

**DOI:** 10.3389/fmed.2022.797586

**Published:** 2022-03-17

**Authors:** Siddhesh P. Thakur, Matthew K. Schindler, Michel Bilello, Spyridon Bakas

**Affiliations:** ^1^Center for Biomedical Image Computing and Analytics, University of Pennsylvania, Philadelphia, PA, United States; ^2^Department of Radiology, Perelman School of Medicine, University of Pennsylvania, Philadelphia, PA, United States; ^3^Department of Pathology and Laboratory Medicine, Perelman School of Medicine, University of Pennsylvania, Philadelphia, PA, United States; ^4^Department of Neurology, Perelman School of Medicine, University of Pennsylvania, Philadelphia, PA, United States

**Keywords:** Multiple Sclerosis, clinical setting, machine learning, deep learning, gadolinium

## Abstract

Multiple Sclerosis (MS) is a demyelinating disease of the central nervous system that affects nearly 1 million adults in the United States. Magnetic Resonance Imaging (MRI) plays a vital role in diagnosis and treatment monitoring in MS patients. In particular, follow-up MRI with T2-FLAIR images of the brain, depicting white matter lesions, is the mainstay for monitoring disease activity and making treatment decisions. In this article, we present a computational approach that has been deployed and integrated into a real-world routine clinical workflow, focusing on two tasks: (a) detecting new disease activity in MS patients, and (b) determining the necessity for injecting Gadolinium Based Contract Agents (GBCAs). This computer-aided detection (CAD) software has been utilized for the former task on more than 19, 000 patients over the course of 10 years, while its added function of identifying patients who need GBCA injection, has been operative for the past 3 years, with > 85% sensitivity. The benefits of this approach are summarized in: (1) offering a reproducible and accurate clinical assessment of MS lesion patients, (2) reducing the adverse effects of GBCAs (and the deposition of GBCAs to the patient's brain) by identifying the patients who may benefit from injection, and (3) reducing healthcare costs, patients' discomfort, and caregivers' workload.

## 1. Introduction

Multiple Sclerosis (MS) is a chronic immune-mediated disease that affects the central nervous system (CNS) with a complex pathophysiology. The prevalence of MS in the United States of America is reported as approximately 1 million adults (1), with several more million patients recorded worldwide.

MRI is an essential tool in the diagnosis and treatment monitoring of MS. Patients with MS typically undergo annual follow up MRI scanning that commonly includes T1 post-contrast images to assess for subclinical disease, i.e., formation of new focal demyelinating lesions in the absence of clinical symptoms. Previous work has shown that in the absence of a new T2-weighted Fluid-Attenuated-Inversion-Recovery (T2-FLAIR) lesion, contrast does not usually add additional clinical information to the interpretation of the scan (2). A common challenge in the clinical assessment of MS is relying on visual interpretation of images, particularly in the case of high lesion burden, to determine if new lesions developed. Automated techniques could aid clinicians in their visualization of new MS lesions improving efficiency and confidence in clinical decisions.

Here, we present a computer-aided-detection (CAD) approach that uses machine learning techniques to detect changes in white matter brain lesions on MRI scans of patients with MS. The presented system has been deployed and fully integrated into the routine clinical workflow for 10 years. Although it was initially designed and used solely as a neuroradiological aid in detecting MS lesion burden changes, this CAD approach is also contributing to reducing the number of gadolinium injections in the MS patient population. Specifically, our CAD system focuses on two targets: (i) assessing MS lesion burden changes from a given previous time-point; and (ii) reducing the administration of GBCAs, based on the detection of new/growing lesions. In a nutshell, the development of our CAD system initiated in 2009 and following its offline evaluation it is clinically translated and integrated in the routine clinical workflow since 2012 focusing solely on its first target. In 2019, the same CAD system was further successfully evaluated and integrated to the clinical workflow for the reduction of GBCA administration.

## 2. Literature Review

**Figure 1:**
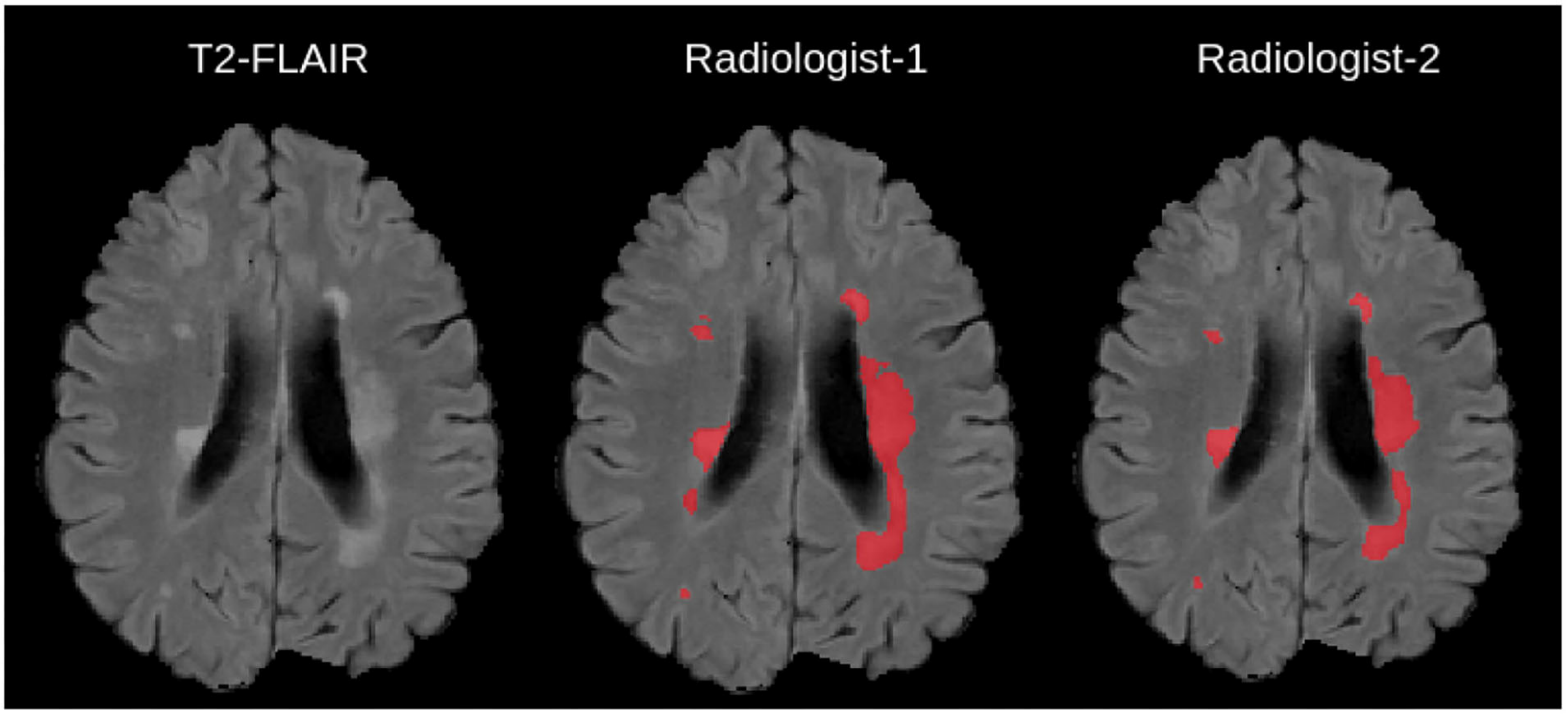
Example Illustration of Multiple Sclerosis lesions overlaid on a 2-D T2-FLAIR scan, together with manual delineations from independent experts taken from (4).

According to related literature (3), computational methods for the assessment of MS, can be divided into two categories: (1) lesion detection, and (2) lesion-change detection. As shown in Figure 1, the lesion detection approach detects both static and dynamic MS lesions on a given single-time MRI volume. These segmentation methods are usually supervised and rely on distinguishing hyperintense lesions from normal appearing white matter tissue in the brain. The lesion-change detection is a longitudinal analysis of volumes taken at different time-points, and a lesion quantification approach is required to see the lesion changes quantitatively (5). A lesion-change occurs as a result of “*tissue transformation*” or even “*tissue deformation*” (6). “*Tissue transformation*” in the context of MS lesions refers to the change in signal intensity within a MS lesion (after accounting for acquisition differences), whereas “*tissue deformation*” refers to surrounding tissue changes as a result of the lesion's expansion or contraction. Neurologists referring patients for a follow-up MRI, want to know if new lesions have formed since the previous timepoint. This information may prompt neurologists to modify the treatment regimen, in order to avoid future recurrences.

The current clinical routine to detect new white matter lesions is based on the visual observation and longitudinal comparison of T2-FLAIR MRI brain scans, by neuroradiologists, from current and previous sessions. However, the typical acquisition protocol for MS patients includes high-resolution 3-D MRI scans, which render this manual reviewing process a tedious and time-consuming task. The current clinical practise based on visual observation can be inaccurate if there are large angulation differences between the two studies or at times, when particular 3-D protocols are not followed and 2-D scans of large slice thickness are acquired instead. These constraints suggest that the utilization of CAD tools could contribute and software-based interventions to speeding up the whole procedure, while at the same time improving the accuracy of quantification. Taking into consideration the optimal patient care, a semi-automatic “*human-in-the-loop*” approach (where the neuroradiologist removes potential false positive detections generated by the computational tool) may be the best solution.

There are multiple ways for MS lesion detection, and some of them are (i) intensity-based approaches, which depend on detecting the changes of intensity (7, 8), (ii) deformation-based approaches, which analyse the deformation of brain tissue (9, 10), (iii) segmentation-based approaches, which segment white matter hyper-intensities from the acquired scans (11, 12), and (iv) subtraction-based approaches, which depend on subtracting two longitudinal scans (7).

In the intensity-based approaches, a voxelwise comparison is made between MRI scans of different time-points to detect and segment new MS lesions (7, 8). In the deformation-based approaches, the new lesions detected in a T2-FLAIR scan are identified by analyzing the deformation fields between the different MRI scans, obtained through non-rigid registration (9, 10). The non-rigid registration method between the two timepoints has shown to improve the detection of the new T2-w MS lesions in longitudinal studies (10, 13). These deformation fields can be generated through non-rigid registration approaches, either based on optimization (14) or newer learning-based approaches (15). Typically, both the “*tissue transformation*” (*via* intensity change) and the “*tissue deformation*” occur, and as such the mass effect of the particular lesion needs to be taken into account for a precise assessment of the lesion's evolution status.

Furthermore, numerous strategies that combine intensity-based and deformation-based approaches have been proposed. Cabezas et al. (10) modified Ganiler et al. (7)'s subtraction pipeline by merging subtraction and Deformation Field (DF) operators to reduce the amount of false positive lesions found by the subtraction pipeline. Registration is characterized as an optimization issue that must be solved for each volume pair of longitudinal scans using a similarity metric, while enforcing smoothness requirements on the mapping in these approaches. Because solving this optimization is generally computationally costly (16–19), it is exceedingly slow in practise. Various GPU-based accelerated methodologies have been presented to improve the efficiency and speed up the optimization (20–22).

Currently, Convolutional Neural networks (CNNs) have shown superior performance in brain imaging, particularly for segmenting tissues, (23, 24), brain extraction (25–27), brain tumors (27–33), and white matter lesions (34, 35). During training, learning-based registration techniques learn a parameterized registration function from a set of images. Some proposed methods (36, 37) use a precomputed DF as the ground truth (GT), while others depend solely on image registration or segmentation masks, without comparing the predicted DF to a precomputed DF (38, 39). Balakrishnan et al. (15) developed a new CNN approach that computes the deformation between two images by training the network using a similarity metric and a regularization term similar to traditional registration methods, yielding results that are comparable to current state-of-the-art approaches.

## 3. Materials and Methods

### 3.1. Data

The routine MRI acquisition protocol for MS patients in the University of Pennsylvania Health System (UPHS) network includes (i) 3-D T2-FLAIR (ii) high resolution isotropic or near-isotropic T1 pre-contrast [3-D magnetization-prepared 180 degrees radio-frequency pulses and rapid gradient-echo (MPRAGE)], as well as (iii) 2D T2-weighted images, and (iv) 30-direction Diffusion Tensor Imaging (DTI). Additionally, 3-D T1 post-contrast images are optional and acquired only if new lesions are detected from the CAD results. All MRI sequences described here are acquired within the UPHS, at multiple satellite sites. However, the vast majority of MS patients get their MRI scans at the main site of the Hospital of the University of Pennsylvania (HUP). The scanner magnetic field strength of the equipment used to acquire these MRI scans was either 1.5 or 3 Tesla, with the HUP scanners being exclusively at 3 Tesla.

The CAD approach presented here uses only the 3-D T2-FLAIR, which is the first acquired sequence in the UPHS acquisition protocol for MS patients. In some rare cases, the prior T2-FLAIR sequence is part of an outside study that has been uploaded to PACS.

Since the acquisition parameters vary across sites, we briefly cover them as majority of scans are performed at 3/1.5 Tesla, with the sagittal 3D T2/FLAIR acquired using the following parameters: TR/TE/TI = 5,000/395/1,800 ms, FOV 250 × 250 × 160 mm, matrix of 256 × 256 × 160, near isotropic 1*mm*^3^ voxel size. Outside studies with only available 2D T2-FLAIR scans with slice thickness larger than or equal to 5mm are not considered useful and as such our CAD system is not applied to them. However, outside studies with 2D T2-FLAIR scans with <5mm thickness are still used by resampling the higher resolution scans to match the lower resolution images. Notably, the proportion of patients with 2D T2-FLAIR scans from outside studies have been rare.

### 3.2. CAD System Overview

**Figure 2:**
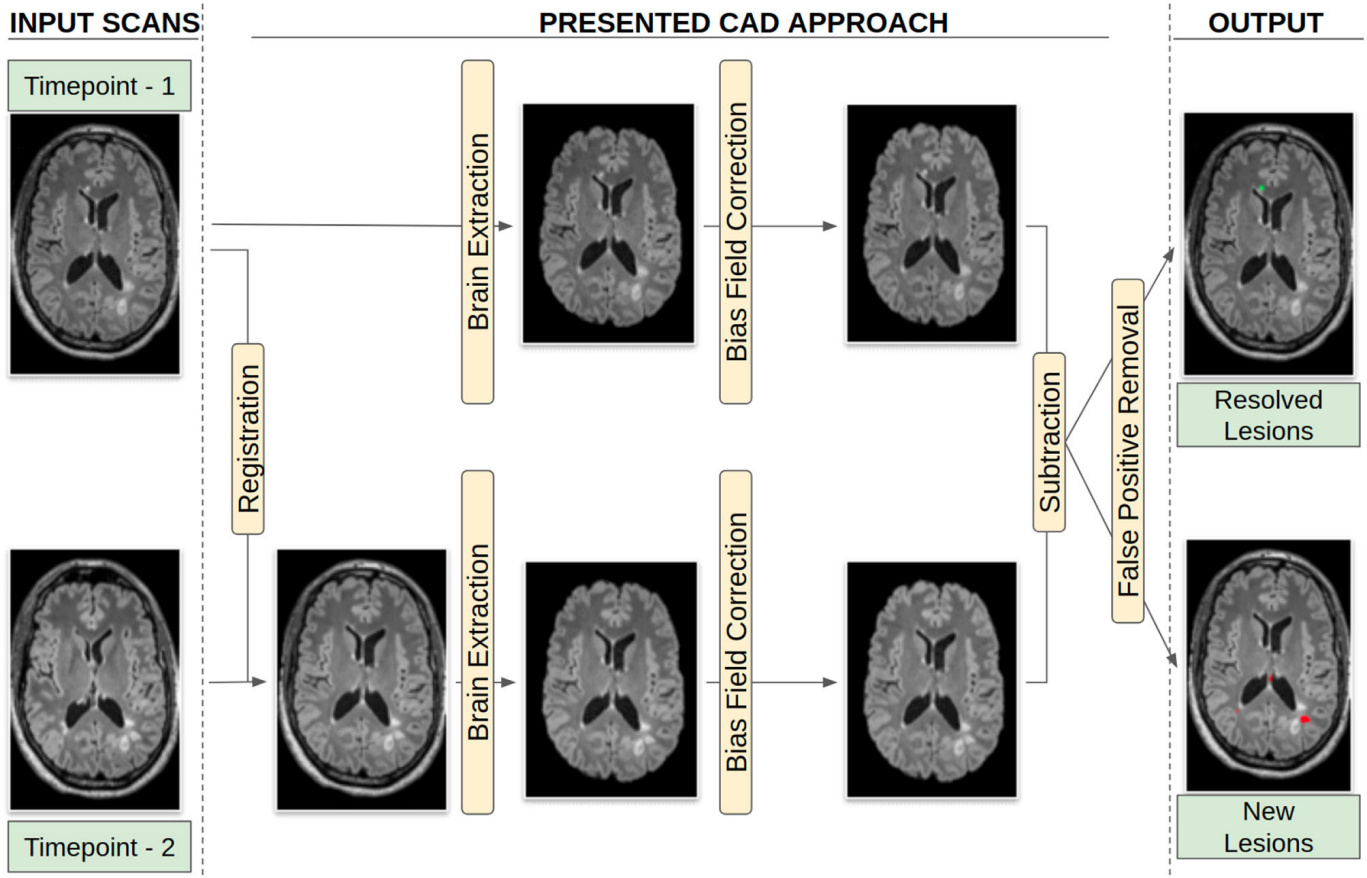
Workflow followed for generating the lesion maps.

The functionality of the CAD approach is described in the following sections and visually summarized in Figure 2. The overview of the CAD method can be explained as follows: after acquiring the 3D T2-FLAIR scan at Timepoint-2, the CAD system is executed. The registration of Timepoint-2 to a Timepoint-1 3D T2-FLAIR scan occurs, followed by brain extraction and bias field correction for both time points. Then subtraction and false positive reduction methods are applied to identify new lesions, as well as resolve false positives generated by the CAD system. While the CAD system is running, for the routine protocol, we acquire the T1 precontrast, 2D T2-weighted, and DTI images. After the CAD system points out whether new lesions are present, the decision to inject GBCAs is delivered to the MRI technologists.

**Figure 3:**
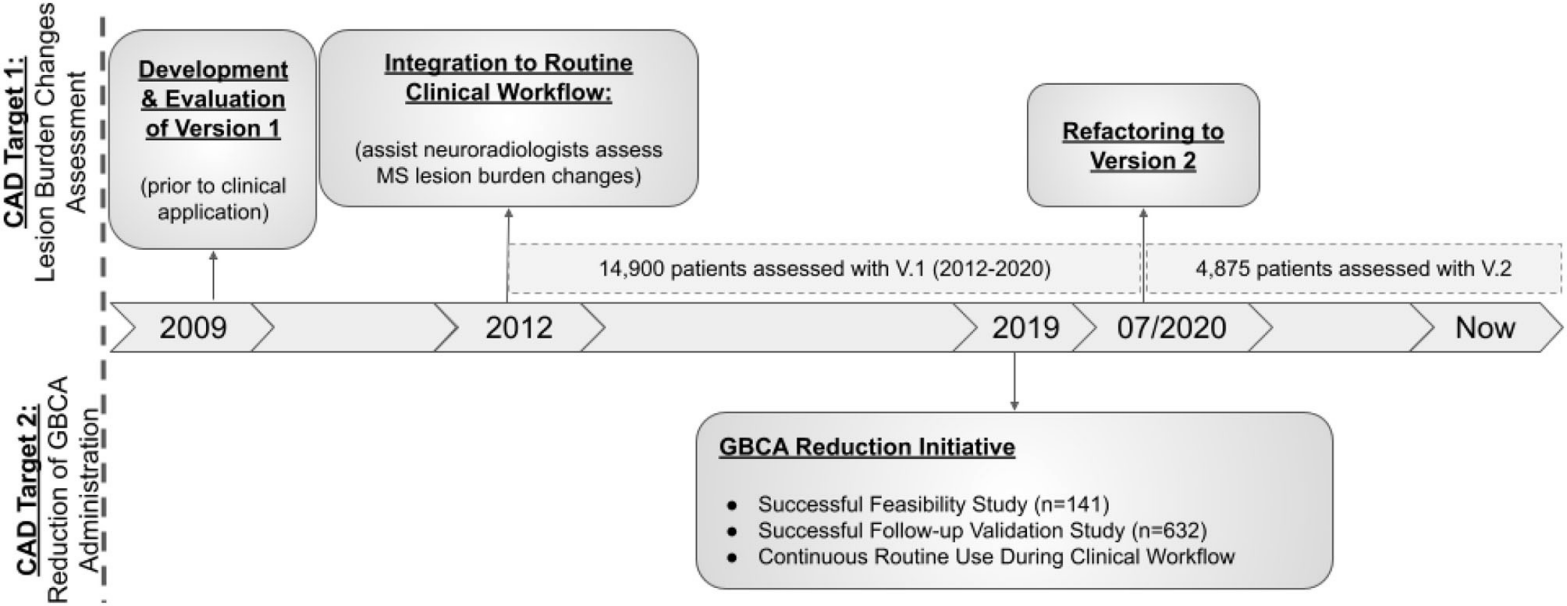
Visual representation of the CAD's lifecycle to-date.

The hereby presented computational approach, integrated and routinely used in clinical practice since April 2012, has been applied to the assessment of MS lesion scans more than 19, 000 times and is currently assessing more than 200 MS patients per month. Figure 3 provides a visual representation of the CAD's lifecycle to-date. The “3-D lab” (i.e., a UPHS team of technologists, trained for executing specific software, e.g., for post-processing cardiac CT, MR arteriograms, and more) currently runs and monitors the CAD approach for every scanned MS patient.

### 3.3. Pre-processing

Prior to any image processing specific to the CAD targets, a set of pre-processing steps are considered essential toward defining the search space to assess lesion burden changes.

All acquired MRI scans are stored in the SECTRA Picture Archiving and Communications System (PACS)^1^, as DICOM file sequences. Once a scan is retrieved from the PACS, the CAD system first converts the DICOM file sequences into the Neuroimaging Informatics Technology Initiative (NIfTI) (40) file format to facilitate subsequent image processing steps.

Since the CAD approach is intended for use across multiple UPHS sites, some harmonization needs to be considered in the imaging space to account for any heterogeneity in the acquisition protocol, thereby ensuring consistent interpretation of the input scans. The most typical harmonization considered here is on the normalization of the scanning resolution, since the acquired MRI scans can range from good quality 1*mm*^3^ isotropic resolution (or near-isotropic) to a scan of much higher slice thickness, e.g., 5*mm*^3^. Specifically, when the resolution of the scans across the two time-points differs, the higher resolution scans are downsampled to match the lower resolution scans. The reason to select the lower resolution target is to reduce interpolation artifacts that would have been generated when going from lower resolution to higher resolution. The software can work with 2D images, but the low out-of-plane resolution (slice thickness) limits results accuracy.

Following this resolution normalization, all apparent non-brain tissue has to be removed from the MRI scans to facilitate optimal downstream analyses, by removing parts of skull and to keep the region of interest focused to the brain. Firstly, each patient's Timepoint-2 T2-FLAIR MRI scan has to be rigidly registered to the patient's Timepoint-1 T2-FLAIR anatomical space. Then, the step of brain extraction (also known as skull-stripping) is performed, in order to reduce false positives that may be generated during the downstream analysis, by including portions that do not belong to the brain tissue.

### 3.4. Intensity Processing

**Figure 4:**
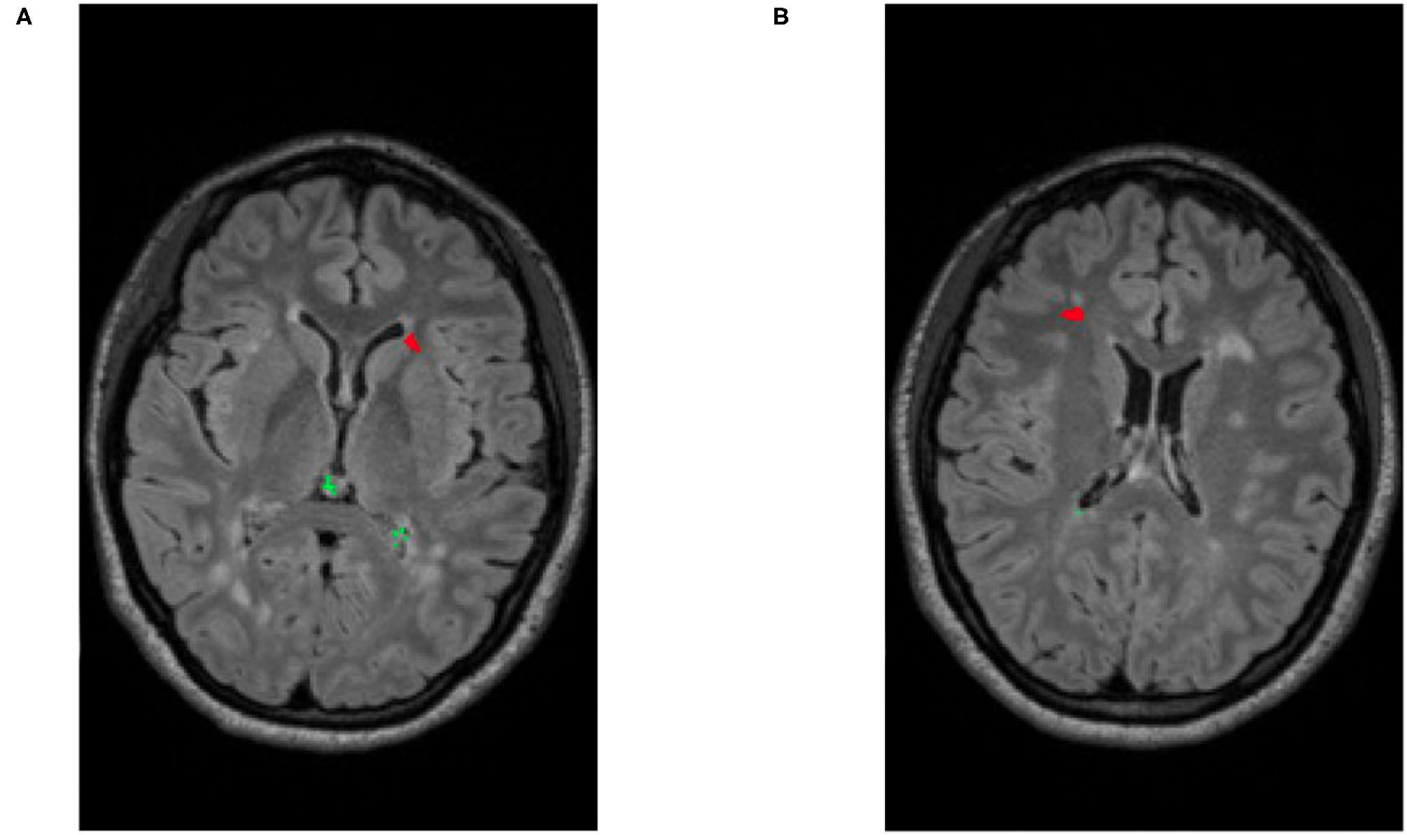
Illustrative examples of detecting false positives and new lesions. **(A)** An example of patient with two identified false positives (depicted in green) and a new lesion (depicted in red). **(B)** Example of a different patient with a single identified false positive (green) and a single new lesion (red).

After identifying the complete search space comprising of the brain tissue apparent in the acquired scans, certain intensity processing needs to take place, to facilitate further analyses. Firstly, the magnetic field strength inhomogeneties observed in the acquired scans are corrected by applying the N4 bias field correction (41), available through the ANTs Toolkit (42). We then apply histogram matching to normalize intensities between the “Timepoint-1” and “Timepoint-2” scans, prior to the subtraction process. This step allows us to take into account any contrast differences that are not related to the lesion appearances. Subsequently, an intensity subtraction takes place between the different time-point MRI scans (Timepoint-1 & Timepoint-2). This subtraction operation identifies new lesions (i.e., through their post-subtraction higher intensity appearance). Thirdly, and importantly, a false positive reduction routine is applied to compensate for false positive “artifacts” occurred due to potential misregistrations. This routine leverages the temporal intensity information between the T2-FLAIR scans of Timepoint-1 and Timepoint-2. Specifically, it assesses the pixel intensity of the lesion's center of gravity, and if it is higher in Timepoint-1 the identified lesion is considered as a false positive, but otherwise a true positive. If this criterion is not met, the identified lesion is considered as a false positive. However, in order to maintain high sensitivity, false positive detections from the CAD are tolerated, and are eventually discarded by human evaluation. An example of a true lesion and a false positive detection is shown in Figure 4.

### 3.5. Atlas Mapping and Lesion Quantification

**Figure 5:**
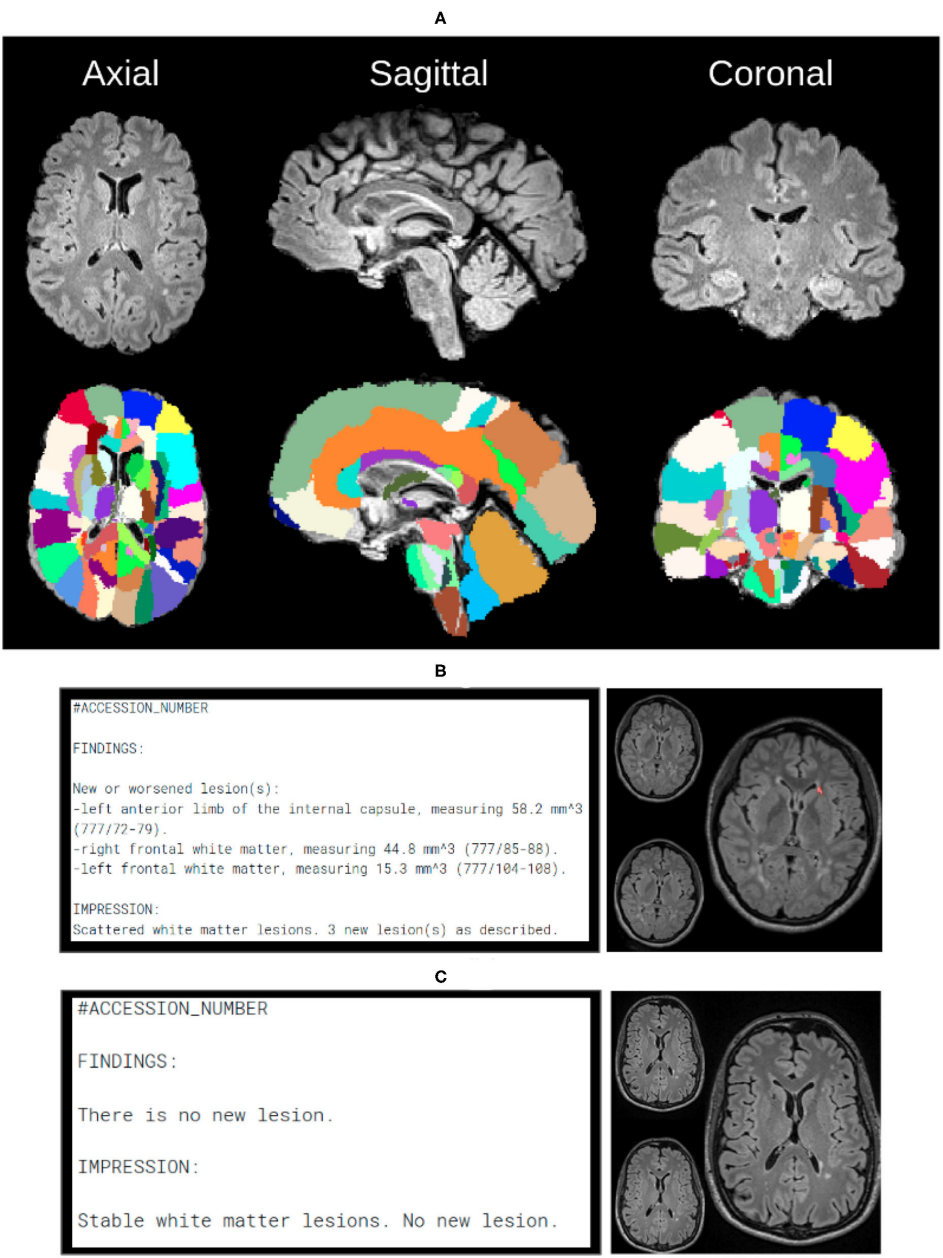
In order to create automated reports, we need anatomical atlas, which can be seen through the subfigures. **(A)** Over 130 anatomical regions of jacob atlas identified and overlayed which allows the software to detect the exact location of new lesions in the brain. **(B)** Example of the automatically generated report, indicating new found lesions. **(C)** Example of the automatically generated report, indicating lack of no newly identified lesions.

After any new or changed lesions are detected, the lesion space is affinely registered to the Jacob Atlas Map (43) (Figure 5), to identify an approximate anatomical location of the lesion. Additionally, the size of new lesions is also returned in *mm*^3^. These measurements are included in a draft radiology report available to the neuroradiologist for editing, thereby improving patient care with quantification information, and improving radiology workflow efficiency. Figures 5B,C depict representative positive and negative report examples, respectively.

**Figure 6:**
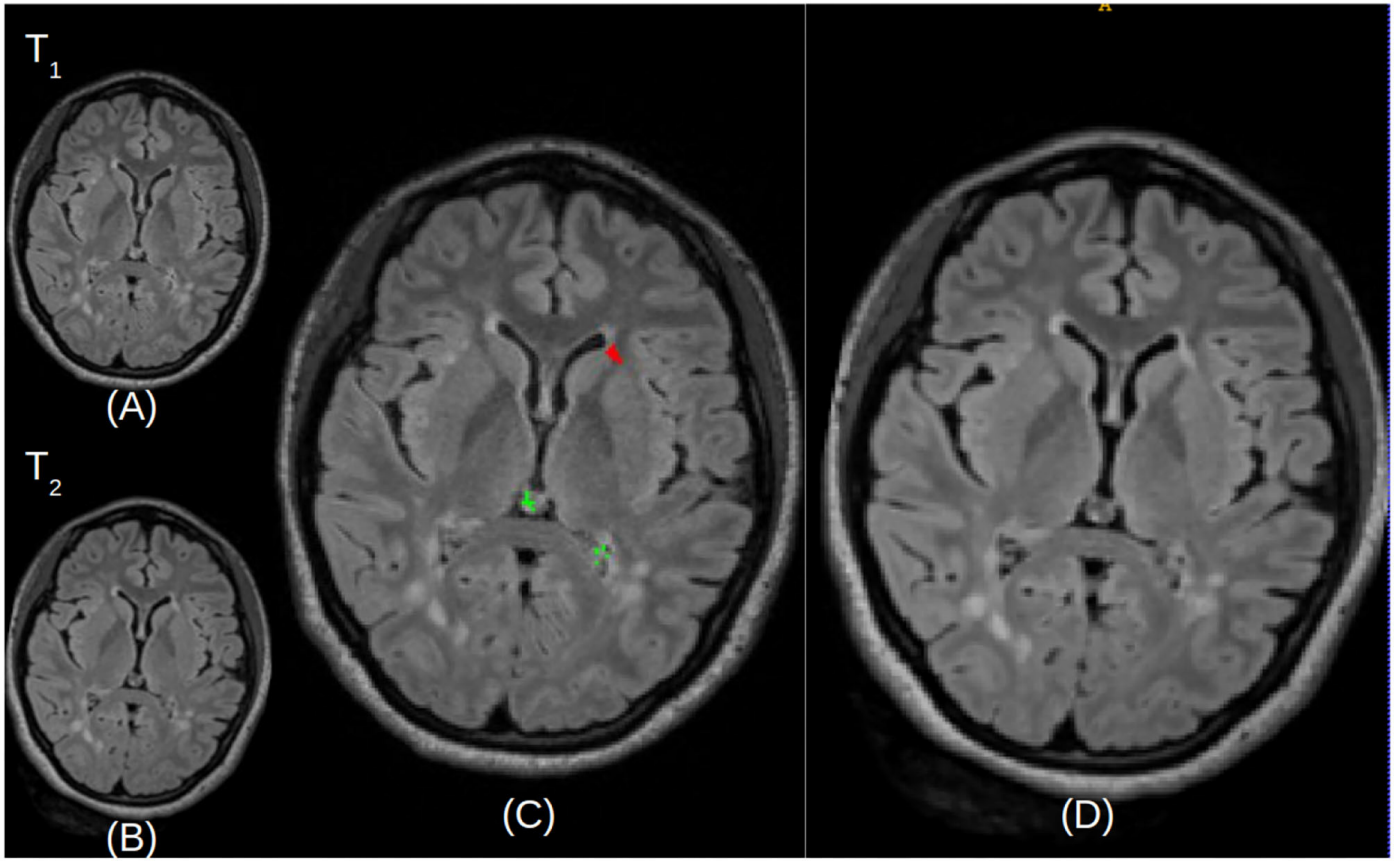
Illustrative examples of resulted images stored in the DICOM file format. **(A,B)** Represent the Timepoint-1 and Timepoint-2 scans of a given patient, respectively. **(C)** Describes Timepoint-2 **(B)**, with superimposed annotations for detected new lesions (depicted in red) and false positives (depicted in green). **(D)** Is a larger version of Timepoint-2 **(B)** without annotations, for visualization purposes.

Finally, the CAD system generates DICOM files from the lesion map images, with the temporal pair of images adjacent to assist radiologists re-verify the images manually, without the need of opening up images from every Timepoint separately. Figure 6 shows the scans of Timepoint-1 (Figure 6A) and Timepoint-2 (Figure 6B) on the image and the predicted lesions (Figure 6C) in the larger size to identify lesions in the subject. These DICOM files are sent to the patient's folder in PACS, so they are available to the neuroradiologist that reads the case.

### 3.6. Gadolinium Reduction Initiative

A new initiative was started in 2019, namely to use the CAD results not only to assist neuroradiologists, but also to determine in real-time which MS patients would benefit from T1 post-contrast imaging. Since the discovery that gadolinium deposition can be detected in the brain of patients that undergo serial MRI studies with GBCAs, many people involved in the domain of Radiology have started initiatives to decrease the numbers of unnecessary contrast injection when performing MRI scans (44). This is one example of application of the principle of precaution, as the long term effects of this deposition in the brain, and probably other organs of the body, are yet unknown and to be determined. The ability of the presented CAD approach to determine accurately and in real time the patients that would benefit from the injection of GBCAs and those that would not, has decreased the rate of injections substantially. This not only has a positive impact on patients by decreasing their exposure to unnecessary gadolinium, but also benefits the caring healthcare institution.

The principle of this initiative is based on the routine use of the CAD system and only in the case of newly detected white matter lesions to intravenously inject GBCAs, enabling the acquisition of post-contrast T1 imaging. Specifically, once the patient is placed in the scanner, the MR technologist initiates the acquisition of the 3D T2-FLAIR sequence. Once this acquisition is complete, the MR technologist contacts the 3D lab technologist to runs the CAD tool. While the CAD tool is being executed, the MR technologist continues with the acquisition of the remaining non-contrast sequences. Once the CAD 3D Lab technologist assesses the CAD results, informs the MR technologist if there is a need for an intravenous injection (e.g., butterfly) and an acquisition of a T1 post-contrast sequence, subject to a new lesion being identified by the CAD system.

To assess the value and the potential clinical relevance of this gadolinium reduction initiative, we conducted a initial 2-month feasibility study involving 141 patients. During this feasibility study, the CAD was already integrated in the clinical workflow for the assessment of lesion burden changes, and hence the feasibility study was directly conducted. After the successful conclusion of this initial feasibility study, we conducted a follow up validation study, including 632 MS patients over the course of 3 months. The purpose of the follow-up study was to confirm the success of the approach in reducing the use of GBCAs, in a larger patient population. The metrics of sensitivity and specificity were calculated for patients who received GBCAs when new brain lesions were found (Equation 1). Following the successful conclusion of both studies, we started using the CAD system as part of our clinical routine for reducing the unnecessary use of GBCAs.


$$\begin{array}{l} sensitivity=\frac{\text{GBCA given for new brain lesion}}{\text{GBCA given for new brain lesion + GBCA not given for new brain lesion}}\left(1\right) \end{array}$$

Inclusion/exclusion of patients in our studies was based on informed consent of participation. For any included patient, the choice to inject GBCA was based on the detection of new lesions as communicated from the CAD operator to the MR technician. The monitored outcome was the performance of correctly identifying patients in need of GBCAs with the use of our CAD system. The sensitivity and specificity calculated here assess the results of the feasibility study (Table 1), as well as the follow up validation study (Table 2) of giving Gadolinium to MS patients when new lesions are detected.

**Table 1:** GBCA reduction initiative: results from the 2 month feasibility study.

	**New brain lesion**	**No new brain lesion**	**Total**
Gad given	14	3	17
Gad not given	4	120	124
Total	18	123	141

**Table 2:** GBCA reduction initiative: results from the 3 month follow up validation study.

	**New brain lesion**	**No new brain lesion**	**Total**
Gad given	119	146	265
Gad not given	17	350	367
Total	136	496	632

### 3.7. CAD's Lifecycle To-Date

Almost 20, 000 patients have been assessed with the clinically deployed CAD system to-date. However, the presented CAD tool has undergone a code refactoring during its lifecycle, to improve performance in terms of execution time and sensitivity, resulting in a second version (Figure 3). Specifically, the initial development and evaluation of the presented CAD tool has successfully concluded in 2012, resulting in its original deployed v.1.0. This version was integrated to the routine clinical workflow for MS patients across the UPHS network. Ever since, we have been monitoring technological developments and methodological advancements that could improve the performance of the deployed tool. Taking into consideration its high throughput application we have only performed some basic code refactoring in 2020, when we observed that the numbers of assessed scans were lowered due to pandemic-related cancellations. The number of patients assessed during the clinical use of v.1.0 (2012-07/2020) and v.2.0 (07/2020-Now) were 14, 900 and 4, 875, respectively.

The algorithmic differences between the CAD tool's v.1.0 and v.2.0 relate to the steps of rigid registration and brain extraction. Further modifications have also been considered that are unrelated to any methodological components and are associated with the optimization of graphical elements of the tool according to feedback from the technologists in the “3D-lab”. For the rigid registration step, we specifically substituted the FSL's FLIRT (45) algorithm that was used in v.1, with “Greedy” (https://github.com/pyushkevich/greedy) (46) to optimize for the total execution time. “Greedy” is a CPU-based C++ implementation of the greedy diffeomorphic registration algorithm (47) and was designed and developed for rapid registration of radiologic scans. “Greedy” shares the Symmetric Normalization (SyN) of the ANTs registration approach (42). Greedy, on the other hand, is non-symmetric, which makes it quicker (in applications like multi-atlas segmentation, where symmetric property is not required). For the brain extraction step, the initial version of the CAD system (v.1.0) used the “Brain Extraction Tool” (BET) (48). During the CAD's refactoring to its second version, in 2020, BET was substituted by an in-house deep learning based method (26) developed explicitly for brain MRI scans including pathologies, with the intention of improving the execution time, as well as the brain extraction quality.

## 4. Results

**Figure 7:**
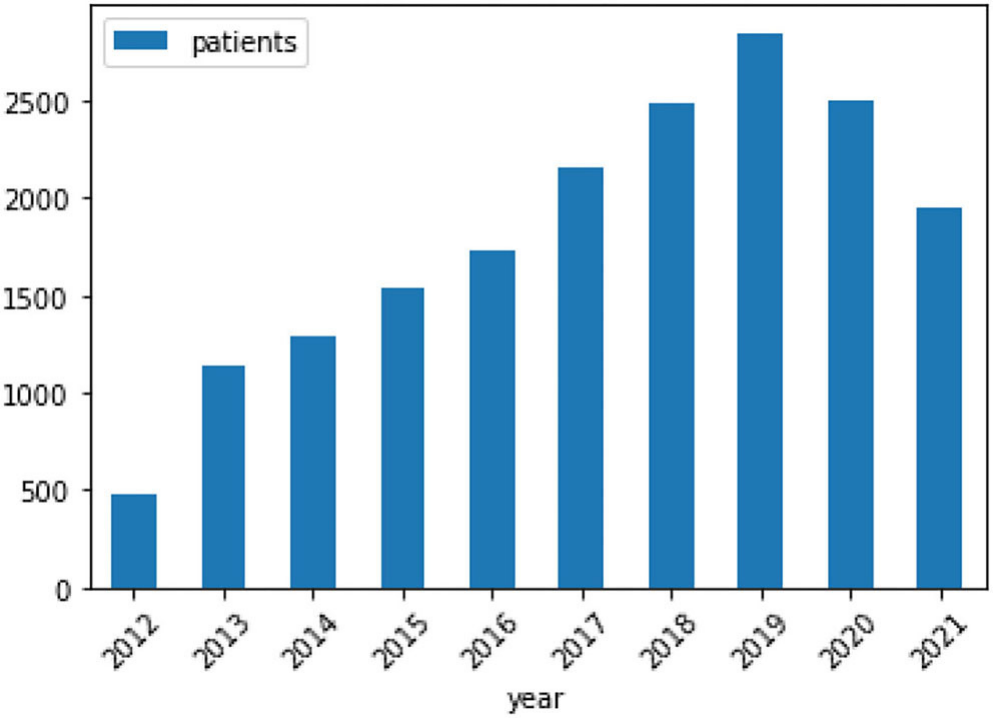
Annual use of the software displayed per year for the number of patients assisted.

Figure 7 depicts the number of clinical cases assessed by the CAD software, since its integration into the routine clinical workflow. The increase over the years reflects both the growth of the patient population at the UPHS MS clinic and the greater application of the CAD software across the UPHS network, i.e., at satellite locations. We should note the drop in the patients evaluated in 2020 due to the COVID-19 pandemic, when patients cancelled or postponed their followup MRI examinations.

For the assessment of lesion burden changes, we have not conducted an explicit quantitative performance evaluation of the two versions of the software. However, the “3D-lab” technologists have internally reported the sensitivity of the initial version in detecting new lesions as 88%, whereas the sensitivity of v.2.0 being greater than 95%, following the aforementioned algorithmic modifications. In terms of execution time, the “3-D lab” technologists require minimal manual intervention to execute the tool (approximately 1 min), and the total time that the approach takes to perform a single patient assessment, on an 4-core CPU (Intel Xeon W-2123 3.60 GHz), has been reported as 11 min for v.1.0 and 5.5 min for v.2.0, on average. The false positives are relatively easy to discard by humans because they tend to occur outside of the white matter of the brain, and in specific areas of the images that are inherently noisy.

For the initial testing phase of gadolinium reduction initiative, we conducted a 2 month feasibility study during which a total of 141 patients participated. Following Table 1, we note an 88% reduction in the overall use of GBCAs. We further note a 98% reduction of GBCAs use, when considering only patients with new lesions. Following a performance evaluation, we maintained a high specificity (shown in Table 3), while keeping high sensitivity of 78%. This feasibility study showed promising results, and the new protocol, with contrast imaging conditioned upon finding new disease activity on CAD results, became the current standard.

**Table 3:** GBCA reduction initiative: quantitative performance evaluation from the feasibility and the follow up validation studies.

**Metric**	**Feasibility study**	**Follow-up study**
Sensitivity	0.78	0.88
Specificity	0.98	0.71
Precision	0.82	0.45
Recall	0.78	0.88
Positive predictive value	0.82	0.45
Negative predictive value	0.97	0.95
False positive rate	0.02	0.30
False negative rate	0.22	0.13
Accuracy	0.95	0.75
F1 score	0.80	0.59

Further evaluation of the gadolinium reduction initiative described a 3 months analysis for 632 additional MS patients, as a follow up validation. In this analysis, we note a reduction of 58% of GBCAs' use, which is lower when compared to the 88% reduction observed in the feasibility study. We further note a 71% reduction of GBCAs use on only patients with existing lesions. This study yielded an increase in sensitivity to 88% (from 78% of the feasibility study), while a reduction occurred in specificity from 98% of the feasibility study to 71%.

These metrics are being passively tracked by the “3-D lab”, and the current estimate is about 85% reduction in GBCAs use, as the protocol continues to be utilized across nearly all UPHS sites.

## 5. Discussion and Future work

In this study we have presented a CAD based method deployed and integrated to the routine clinical workflow for (i) assisting neuroradiologists assess MS lesion burden changes, and (ii) reducing the need for use of GBCAs. We demonstrate the successful evaluation of this computational approach in both the initial evaluation studies and during its routine clinical use, following its complete integration to the clinical workflow since 2012. The findings of this study support our claims that CAD based systems built around clinical settings for MS can contribute in improving patient care and assist radiologists in making better informed decisions.

Temporal changes in patients with existing diagnosed MS lesions were identified better through the presented computational approach using a 3-D T2-FLAIR MRI sequence, than the routine clinical interpretation based on visual observation. The computational approach assisted in improving the sensitivity and false-positive ratio in identifying patients with new (or growing) lesions compared to manual interpretation. Cases can be run in real time (during the patient scanning session, and in < 10 min) within the clinical workflow due to the processing time being so short. The approach has been tuned toward producing the highest possible sensitivity of 90% on a patient basis, where GBCAs are given only when necessary, but still with a low rate of false positive of 30%, allowing for efficient temporal change assessment (Table 3).

Non-enhancing new lesions are also of great interest from a clinical standpoint. In fact, as the number of treatment options for MS patients grows, neurologists caring for them are more interested than ever in knowing if new lesions have emerged from previous scans, regardless of their enhancing status. The presented computational approach is not intended to detect lesions that are enhancing. However, we do not believe that these are clinically significant, and the detection of enhancing lesions “manually” (i.e., by visual observation) is relatively simple.

The presented computational approach helps answer the essential clinical question that neurologists are asking when ordering a follow up MRI scan: “Are there new white matter lesions from the prior scan?”. This is still one of the most relevant metric for assessing the performance of a therapeutic regimen. The high sensitivity (90%) of this approach in detecting new focal MS lesions allows neurologists to determine if a patient's current Disease Modifying Therapy (DMT) is appropriately controlling their disease (i.e., no new MS lesions) and may be continued, or if it is not controlling their disease (i.e., new lesions are detected) and a change in DMT may need to be considered. This is more relevant nowadays that the number of available therapeutic options has increased in the past several years, including higher efficacy drugs that also carry the potential for more adverse effects.

Although the presented approach has a clear benefit to clinical practice, it also has its limitations. One of them is that the different time-point MRI scans have to be acquired at the same institution, or more specifically to have a record stored under the institutional PACS. This is required for the approach to produce appropriate results shared with the attending clinician through the platform used typically for the assessment of MS patients. Use of multi-institutional data with medical record number varying across patients and scanning sessions has not been utilized yet, as it was out of scope of this 10-year analyses. Another limitation is the assessment of the rarely observed spinal cord lesions that are not taken into consideration. Any new lesions formed around the spinal cord are currently not considered/processed, and can be potentially missed through the computational approach, since we have primarily focused only on the brain. Limitations also occur when a lower resolution space is used as the reference space to avoid the interpolation artifacts that are generated going from a lower resolution space to a higher resolution space.

The presented approach could also be further utilized in a clinical research setting, such as drug trials, when the ability of the approach to detect temporal changes consistently and reliably, with high sensitivity 90%, is critical. Although the approach presented here does not calculate either the exact volume of each lesion, or the total change in lesion load, this quantification capability belongs to the immediate future work incorporating multi-institutional pilot projects.

## Data Availability Statement

The datasets presented in this article are not readily available because since the dataset collected contains patient health information, this data cannot be made public. Requests to access the datasets should be directed to the corresponding authors.

## Ethics Statement

The studies involving human participants were reviewed and approved by Institutional Review Board of the University of Pennsylvania. The patients/participants provided their written informed consent to participate in this study.

## Author Contributions

MB and SB: study conception and design. MB, ST, and SB: software design and development. ST, MB, MS, and SB: data analysis and interpretation and reviewed and edited. ST, MS, and MB: wrote the initial manuscript. All authors contributed to the article and approved the submitted version.

## Funding

Research reported in this publication was partly supported by the National Cancer Institute (NCI) and the National Institute of Neurological Disorders and Stroke (NINDS) of the National Institutes of Health (NIH), under Award Numbers NCI:U01CA242871, NCI:U24CA189523, and NINDS:R01NS042645.

## Conflict of Interest

The authors declare that the research was conducted in the absence of any commercial or financial relationships that could be construed as a potential conflict of interest.

## Publisher's Note

All claims expressed in this article are solely those of the authors and do not necessarily represent those of their affiliated organizations, or those of the publisher, the editors and the reviewers. Any product that may be evaluated in this article, or claim that may be made by its manufacturer, is not guaranteed or endorsed by the publisher.

## Footnotes

^1^ https://medical.sectra.com/
